# HIV, malaria and beyond: reducing the disease burden of female adolescents

**DOI:** 10.1186/1475-2875-4-2

**Published:** 2005-01-10

**Authors:** Loretta Brabin, Bernard John Brabin

**Affiliations:** 1 Academic Unit of Obstetrics & Gynaecology & Reproductive Health Care, Research floor, St. Mary's Hospital, Whitworth Park, Manchester M13 OJH, UK; 2 Child and Reproductive Health Group, Liverpool School of Tropical Medicine, Pembroke Place, Liverpool L3 5QA, UK; 3 Emma Kinderziekenhuis, Academic Medical Centre, University of Amsterdam, The Netherlands

## Abstract

In sub-Saharan Africa the highest overlap between malaria and HIV infections occurs in female adolescents. Yet control activities for these infections are directed to different target groups, using disparate channels. This reflects the lack of priority given to adolescents and the absence of an accepted framework for delivering health and health-related interventions to this high-risk group. In this paper it is argued that female adolescents require a continuum of care for malaria and HIV – prior to conception, during and after pregnancy and that this should be provided through adolescent services. The evidence for this conclusion is presented. A number of African countries are commencing to formulate and implement adolescent-friendly policies and services and disease control programs for malaria and HIV will need to locate their interventions within such programs to ensure widespread coverage of this important target group. Failure to prioritize adolescent health in this way will seriously limit the success of disease control programs for malaria and HIV prevention.

## Background

The current generation of adolescents – over one billion – is the largest in history [1] and, far from representing a picture of health [2], many will suffer untimely disease and death. HIV and malaria are responsible for much of the disease burden affecting female adolescents, who suffer disproportionately from these combined infections relative to other age groups. This is due to a high HIV incidence during the period when many adolescents become pregnant for the first time – an event which greatly increases their susceptibility to *Plasmodium falciparum *malaria [3]. Biological interactions between malaria and HIV in pregnancy complicate therapy, which can also be compromised by inappropriate adolescent health-seeking behaviour. The public health importance of HIV and malaria synergism is only now emerging. It has led to a recommendation by the World Health Organization (WHO) that the two disease programs should collaborate to ensure integrated service delivery, especially within the framework of reproductive health services [4]. A case has also been made for linking disease control programs as a more efficient and effective way for lowering the malaria and HIV disease burden [5]. Nevertheless, it is difficult to seen how these goals will be attained unless adolescents are accorded a much higher priority by disease control programs. In this paper we consider the justification, as well as the challenges, of using common approaches to improve delivery of HIV and malaria interventions to female adolescents.

### HIV and malaria burden in pregnant and non-pregnant adolescents

**Figure 1:**
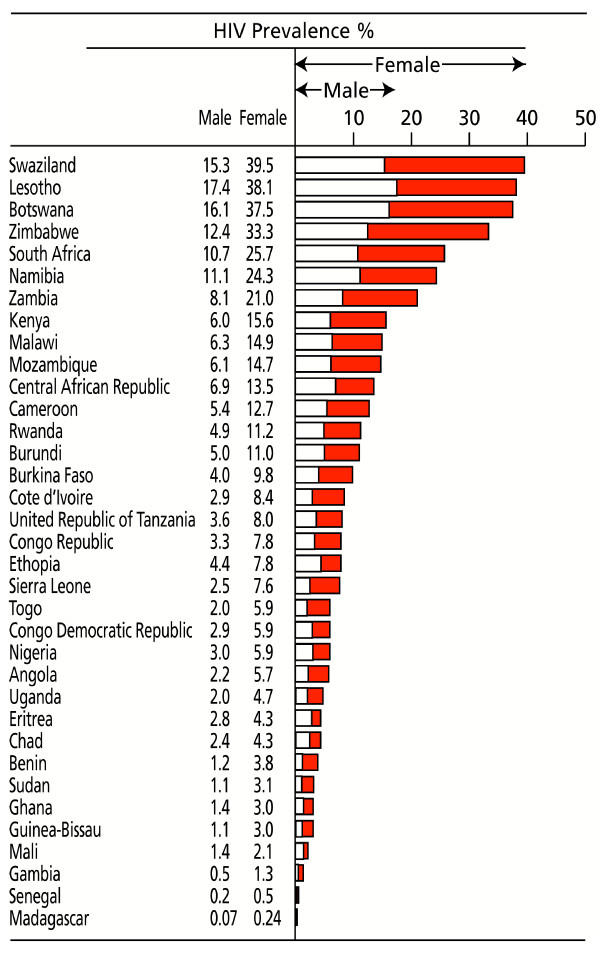
Female and male HIV prevalence among young people 15–24 years of age in sub-Saharan Africa. Compiled from UNFPA^1^

Prevalence estimates for HIV infection [1] for 35 sub-Saharan African countries, for young males and females (15–24 years), are shown in Figure 1. Estimations are often derived from sentinel surveillance of women attending antenatal care (ANC) and tend to overestimate prevalence in adolescents due to the selection of sexually active [6] and less-educated groups [7]. Accepting this bias, in every country, listed HIV prevalence is two to three times higher among females than males. Having an older male partner is associated with a modest increased HIV risk [8] but probably does not explain a sex difference that occurs uniformly across all countries and cultures and at all levels of HIV endemicity. In Rakai, Uganda, only 12.4% of HIV cases in 15–19 year olds was attributed to relationships with men 10 years older [9]. Other sexually transmitted infections (STIs) increase susceptibility to HIV but do not account for the sex difference [10]. Host biological factors may be critical [11]. Sexual maturation takes several years to complete, but menarche is delayed amongst socio-disadvantaged girls, and many will be sexually active before biological maturation is complete. Interventions that delay the onset of sexual activity could reduce exposure at a time of peak biological susceptibility to HIV and highest risk of malaria in pregnancy.

Malaria is an important cause of adolescent hospital admissions in many sub-Saharan African countries with stable malaria transmission [12]. Malaria mortality in African adolescents (both sexes) aged 10–14 years has been estimated at over 45,000 deaths per annum [13]. The highest prevalence of *P. falciparum *infection after childhood is also in young, mostly adolescent, women. Data from Malawi (Table 1) shows that non-pregnant and pregnant adolescents had significantly higher parasite rates than women above 19 years. Primigravidae also have a markedly increased susceptibility to malaria [3]. Young age and nulliparity in the same individual lead to greatly increased malaria risk. This partly relates to reduced acquisition of acquired malaria immunity in young individuals [14] as well as the lack of parity-specific malaria immunity acquired during the first pregnancy [15]. Risk factors for symptomatic malaria in adolescents have not been studied, but both symptomatic and asymptomatic malaria infection will contribute to the development of adolescent and newborn anaemia [16]. HIV infection increases *P. falciparum *prevalence during pregnancy and this is marked during the first pregnancy [17,18], which in Africa, is nearly always in adolescence.

**Table 1:** Malaria prevalence in non-pregnant and pregnant adolescents and adults in the Shire Valley, Malawi

**Adolescent**		**Adult**	
**Non-Pregnant**	Pregnant *	**Non-Pregnant**	Pregnant*

**% (n)**	**% (n)**	**% (n)**	**% (n)**
41.4(29)†	45.9 (122)‡	20.3 (74)	35.4 (345)

* At delivery. Includes women who had received anti-malarials during pregnancy

† Difference with non-pregnant adults χ^2 ^= 4.8; p = 0.028

‡ Difference with pregnant adults χ^2 ^= 4.3; p = 0.04

### HIV and malaria interventions during adolescence

For both HIV and malaria there are effective interventions for disease control. HIV and malaria frequently occur in the same African populations and the same individuals, yet control activities are directed to different target groups, using different channels. Adolescent HIV prevention, with its focus on behavioural change, has taken place largely outside conventional health settings. Few HIV strategies have been directed at pregnant adolescents and/or their partners – an omission noted by a WHO Technical Consultation on married adolescents [19]. In some African countries, by the age of 19 years, half of all female adolescents were in marital or permanent consensual unions [9] that will almost certainly lead to pregnancy. In contrast, malaria control efforts for women have focused on pregnancy, but not on adolescents and certainly not on adolescents before they become pregnant. As a result, in many sub-Saharan African settings, adolescents are often parasitaemic and anaemic when they first become pregnant. The problem is that currently no overall strategy for adolescent health has been approved for most African countries. The burden of infection and disease among adolescents is of sufficient magnitude that a way must be found to provide services to this population. The existence of an appropriate strategy would allow both HIV and malaria interventions to be delivered within a common framework.

In traditional cultures, menarche often marks the transition from childhood to adulthood because it signifies reproductive potential. Nevertheless, adolescence is a physical process that takes a number of years and is usually completed by the late teens. This developmental process should be encompassed within health care provision. Pregnancy may punctuate that process, but during it, the adolescent continues to grow physically and mentally [20]. Strategies to reduce adolescent malaria or HIV should, therefore, provide a continuum of care – before conception, during and after pregnancy – encompassing appropriate information, preventive and curative services. For prevention, adolescents need access to condoms and bednets, information on how to use them and, when and where to go to deal with infection. Services must be available for infection monitoring and treatment, including HIV testing and drug therapy for malaria, anaemia and HIV-related opportunistic infections.

System improvements may not be strictly about adolescent health services, but about support for ANC. Adolescents need good access to pregnancy care and this includes attendance for early pregnancy assessment and screening for HIV and other sexually transmitted infections (STIs). ANC presents an opportunity for HIV prevention, support for discordant couples and care and referral for HIV-infected adolescents. Early assessment is important as the HIV positive individual needs to plan for anti-retroviral treatment (ART) at delivery to prevent mother-to-child transmission. It is also important for malaria control in pregnancy. Intermittent preventive antimalarial treatment (IPT) is the key malaria prevention policy that improves birthweight outcomes and reduces pregnancy anaemia [21]. Its effectiveness depends on the prevalence of HIV infection [22]. It also depends on adherence to the treatment regimen. Coverage with the standard two dose intermittent sulphadoxine-pyrimethamine preventive malaria treatment was lower in younger adolescents in the Shire Valley, Malawi ≤ 17 years, 35.1% versus 18–19 years, 53.7%; p = 0.028, Verhoeff, personal communication), and more than half of all pregnant adolescents received inadequate antimalarial treatment. This demonstrates the importance of pre-conceptual malaria health education for girls. Adolescent mothers need instruction on how to recognize and respond to infant malaria infection and encouragement for using bednets for themselves and their babies.

A major challenge for malaria control is to improve adolescent health prior to the first pregnancy. For non-pregnant adolescents there are several conundrums. On account of drug resistance, malaria combination therapy with artemisinin derivatives is being introduced into an increasing number of sub-Saharan African countries. Artemisinin may be foeto-toxic and should be avoided, except for symptomatic infections with multi-drug resistant malaria, in the first trimester of pregnancy [23]. Inadvertent use or self-treatment by adolescent girls could frequently occur in early pregnancy, before they recognise their pregnancy. The issue of pregnancy testing adolescent girls before treatment with potentially foeto-toxic drugs will need to be considered.

A further issue is that the benefits of IPT extend to pregnant but not nulliparous adolescents who may be anaemic or parasitaemic at the start of their pregnancy. The use of permethrin-treated bednets would protect against anaemia and parasitaemia during the first trimester, but adolescent pregnant girls and their newborns are the least likely to be net users [24].

### Reaching the target population

The growing literature on adolescent-friendly health services shows that adolescents have substantial concerns about the delivery of interventions through health services [25]. Almost universally, adolescents complain of lack of information, confidentiality issues and judgmental attitudes of service providers. There may be barriers of physical inaccessibility, service fees and non-supportive community attitudes. A WHO consultation in Africa concluded that adolescents had "a right to access health services that can protect them from HIV/AIDS and from other threats to their health and well-being, and that these services should be made adolescent friendly" [26]. A WHO Global Consultation on Adolescent Friendly Health Services stated that government ministries should take appropriate action to ensure service provision for adolescents, taking into account cost, epidemiological factors and national health priorities [27]. The role of non-governmental organizations and the private sector in spearheading adolescent projects was acknowledged, but alone, these organizations cannot obtain sufficient coverage of the adolescent population to substantially reduce the HIV and malaria burden. Governments and disease control programs must also consider training health care providers and modifying service delivery, so as to reduce barriers to adolescent uptake.

The only country in Africa that has scaled up adolescent health services is South Africa [28]. The South African National Adolescent-Friendly Clinic Initiative has strengthened the public health sector's ability to respond to adolescent health needs by building capacity and establishing good clinical services which, eventually, will be maintained by district and provincial health authorities. It has demonstrated that an adolescent strategy is achievable if there is political will. The South African approach may not be suitable or affordable for lower income countries. Well-focused services, training of key staff at selected sites, outreach preventive services and good quality, adolescent-oriented ANC are potential service reconfigurations that could be used to extend coverage and increase uptake of both HIV and malaria interventions without creating a separate adolescent service.

What about young men? Available evidence suggests that young men are not attracted to clinic services which are perceived as "female "spaces [29]. In Ghana, for example, 76–89% of all adolescent clinic users were female [30]. Malaria is a reproductive health issue for females, but not for males, and the disease burden of both malaria and HIV infections is much lower in young males. Adolescent females require more health-service based care than males, and this should be reflected in service configuration.

### Creating an enabling environment to promote adolescent access to HIV and malaria interventions

Behavioural change, including appropriate health-seeking practices, is essential for disease reduction. A decline in HIV prevalence amongst adolescents has been reported from Zambia [7], Uganda [31,32] and Tanzania [33] and is attributed to changes in sexual behaviour. In Zambia, between 1996 and 1999, urban adolescent males reported less frequent sexual activity, fewer partners and were more likely to have used a condom at the last sexual encounter. Changes in these indices were less marked, or even deteriorated, among rural female adolescents [7]. Whether behaviour change is the result of HIV programme interventions or other factors is unclear. Recent surveys in sub-Saharan Africa reported that higher female education was associated with lower HIV prevalence and lack of schooling with higher HIV incidence [6] and lower condom use [34]. In western Uganda the proportion of illiterate women fell from 41.6% to 24.6% over six years and may have facilitated behavioural changes, but this link has been little investigated. Illiterate females are likely to be young and married, and to be in a situation where they cannot reduce frequency of intercourse, negotiate condom use or delay having children [19]. In a large pregnancy study in southern Malawi, 73% of 469 nulliparous adolescents were illiterate [35]. They made significantly fewer ANC visits, were less likely to have a supervised delivery and had a higher risk of low birthweight. Reducing illiteracy and increasing educational and vocational opportunities for female adolescents may be critical enabling actions to promote risk reduction, to enhance their social status and to decrease female vulnerability. [36]

### Developing policies to support HIV and malaria control for female adolescents

Few developing countries have well-defined policies for delivering care to the non-pregnant or pregnant adolescent, especially for sexual health. Existing malaria guidelines also do not mention adolescents specifically [12].

An important aspect is the issue of the adolescent's right to sexual and reproductive information and services vis à vis the legitimate rights of parents to act in the best interests of a child, and the health care provider's right to work within the law. These are issues of consent and confidentiality for adolescents considered "minors" and under parental control, or wives under their husband's or husband's family's jurisdiction. When these issues are ignored, adolescents cannot be assured of confidentiality, and health workers are neither bound, nor protected by, the legal system. Rights established in law, or by signed Conventions (eg Convention of the Rights of the Child), may not be implemented. Health policy falls down because it is not explicit about the vulnerability of female adolescents and does not challenge cultural and social norms that can perpetuate the disease burden. Similarly, encouragement for improving female literacy and education may be lacking, reducing the supportive environment for promoting uptake of adolescent health services and safer sex. Advocacy is essential to co-ordinate law, custom and health practice. At another level, it is important to acknowledge that interventions for female adolescents raise ethical issues that are avoided when they are excluded from care. The policy of avoiding treatment of adolescent girls (and adult women) because they might be in the first trimester of pregnancy, raises issues of gender discrimination and has been a serious issue for parasitic disease control programs [37]. Reducing the necessity for first trimester treatments for malaria can be achieved by increasing efforts to redress the malaria burden prior to pregnancy, but this requires reconfiguring service delivery. HIV-positive adolescents who are at the start of their sexual and reproductive lives might be prioritized to receive anti-retroviral therapy, but their relative poverty and lack of social status may lead to their exclusion from access to this intervention.

## Conclusions

For both HIV and malaria, although interventions are available to reduce the disease burden, adolescents are not prioritized. Effective policies and services are needed to reduce the substantial disease burden they experience from these infections. To secure this goal, concerted action across HIV and malaria disease control programs is required. This will reinforce support for adolescent programs rather than weakening them by multiple separate activities, which can confuse implementation. A schedule of activities is required that is reinforced by other systems (e.g. education) to facilitate embedding it into a way of life. These issues need to be addressed head-on by the adolescent health community to achieve the focus needed, and to establish the realistic steps forward. In the short term, developing services to deliver HIV and malaria interventions can act as a catalyst for addressing broader adolescent health service needs. In the medium-term adolescent-friendly health services provide the required platform for the evaluation and delivery of future vaccines and drug therapies for HIV, malaria [38] and other diseases. In the longer term an inter-sectoral adolescent strategy provides a basis for breaking the cycle of maternal-child ill health.

## Authors' Contributions

Both authors made substantial contributions to the analysis, interpretation, drafting and revision of this article. Both have agreed to authorship.
